# Baboons at a Crossroads: Hybridisation Events and Genomic Links of Central Mozambique's Baboons With *Papio* Neighbors

**DOI:** 10.1002/ajpa.70082

**Published:** 2025-06-28

**Authors:** Matteo Caldon, Giacomo Mercuri, Giacomo Mutti, Maria Joana Ferreira da Silva, Felipe I. Martinez, Cristian Capelli

**Affiliations:** ^1^ Department of Chemistry, Life Sciences and Environmental Sustainability University of Parma Parma Italy; ^2^ Barcelona Supercomputing Centre (BSC‐CNS) Barcelona Spain; ^3^ Institute for Research in Biomedicine (IRB Barcelona), The Barcelona Institute of Science and Technology Barcelona Spain; ^4^ BIOPOLIS Program in Genomics, Biodiversity and Land Planning CIBIO Vairão Portugal; ^5^ CIBIO, Centro de Investigação em Biodiversidade e Recursos Genéticos, InBIO Laboratório Associado, Campus de Vairão, Universidade do Porto Vairão Portugal; ^6^ ONE‐Organisms and Environment Group School of Biosciences, Cardiff University Cardiff UK; ^7^ Escuela de Antropología Facultad de Ciencias Sociales, Pontificia Universidad Católica de Chile Santiago Chile

**Keywords:** baboons, Central Mozambique, hybridisation, non‐invasive genomics

## Abstract

**Objectives:**

Hybridisation plays a critical role in species evolution and is widespread among primates, particularly in the genus *Papio*. Several baboon hybridisation zones have been identified in Africa, with Gorongosa National Park in Mozambique being notable for chacma baboons exhibiting phenotypic and genomic traits of both chacma and yellow baboons. This study builds on earlier research by leveraging new genomic data to refine our understanding of the relationships between Central Mozambique baboons and other baboon populations, focusing on chacma, yellow, and kinda baboons.

**Materials and Methods:**

We analyzed uniparental genetic markers alongside autosomal and X chromosome variants, incorporating unpublished low‐coverage genomes from fecal samples collected in Central Mozambique. These data were compared with the broader genomic landscape of *Papio* baboons based on recent surveys.

**Results:**

The analysis of uniparental markers suggests a time to the most recent common ancestor of less than 200kya for chacma baboons in Zambia and Gorongosa, with both lineages sharing a node with yellow baboons from Tanzania less than 1 Mya. Genomic analyses indicate introgression in Central Mozambique and Zambia chacmas likely originated from populations closer to eastern rather than western Tanzanian yellow baboons.

**Discussion:**

Our findings reveal yellow baboon introgression in Central Mozambique chacmas, confirming this being a region hosting baboons with complex ancestry composition. Broader genomic surveys across Mozambique are necessary to uncover the population structure and evolutionary history of chacmas in this area, as well as the role of this region as a biodiversity crossroads for primates.

**Summary**

- Signatures of hybridisation in *P. ursinus* in the north suggest a source more similar to eastern than western Tanzania, *P. cynocephalus* , while *P. kindae* contributions differ across *P. ursinus* populations.
- Analysis of uniparental inheritance systems (mitochondrial DNA and Y chromosome) places the TMRCA between Zambian and Mozambican *P. ursinus* populations at around 170Kya.

## 1 Introduction

Hybridisation is a natural process that involves the interbreeding of two genetically distinct lines, producing offspring (Cortés‐Ortiz 2017; Taylor and Larson 2019). Although it was once met with considerable skepticism, especially in the animal kingdom, it has been shown in recent decades to play a very important creative role in evolution as a source of additional variation (Arnold and Meyer 2006). It is estimated that about 10% of primate species have been involved in such events, including humans (Tung and Barreiro 2017; Cortés‐Ortiz 2017; Zinner et al. 2011; Arnold and Meyer 2006; Meneganzin and Bernardi 2023; Jensen et al. 2023). Particularly interesting are the hybridisations involving baboons (genus *Papio*). This genus consists of six species, but the interconnections between them through hybridisation are numerous. Vilgalys et al. (2022) demonstrated that subtle selection against hybridisation can play a significant role in preserving the taxonomic integrity of primates, even amidst frequent interspecific gene flow. This equilibrium is influenced by several factors, including the selective pressures opposing hybridisation, the potential benefits of introgressed ancestry, migration dynamics, and demographic stochasticity. These findings provide a framework for understanding instances of nuclear swamping, that is, the replacement of the nuclear genome of one species by that of another via sex‐biased hybridisation (Zinner et al. 2011), observed in baboons, despite the associated costs of hybridisation. Vilgalys and colleagues investigated one of the most well‐known baboon hybridisation zones, located in the region spanning Kenya and Tanzania, centered in Amboseli National Park, where *Papio anubis* and *Papio cynocephalus* interbreed. However, other hybridisation zones between different baboon species are also known, including the Awash National Park in Ethiopia between *P. anubis* and *P. hamadryas* (Bergman et al. 2008) and in Kafue National Park in Zambia between *P. kindae* and *P. ursinus* (Jolly et al. 2010; Chiou et al. 2021). Particularly relevant in the last mentioned case is the occurrence of individuals with *P. ursinus—P. kindae* hybrid traits that possess a Y chromosome of *P. kindae* origin although mitochondrial DNA is often of *P. ursinus* origin. The prevalence of *P. kindae* Y chromosome in the hybrid zone suggests that *P. kindae* males have a reproductive advantage over *P. ursinus* males (Jolly et al. 2010; Chiou et al. 2021). Other events of hybridisation involving chacma baboons have been detected but not directly observed, such as ancient hybridisation between *P. ursinus* and *P. cynocephalus* in Central Mozambique, particularly in Gorongosa National Park (Martinez et al. 2019; Santander et al. 2022). Interestingly, all the hybridisation zones reported so far are in/around National Parks. It can be speculated that this might reflect a bias in where research on baboons has been conducted, but it might also emerge from the fragmented distribution of baboons outside protected areas as the result of anthropogenic factors (Ferreira da Silva et al. 2018).

The identification of these events and their characterization in baboons in Mozambique makes it possible to investigate the evolutionary dynamics following hybridisation across combinations of species different from those well‐known and extensively investigated in other African areas, offering additional opportunities to test hypotheses about demographic dynamics and selection, positive and negative, shaping diversity in hybrid populations (Vilgalys et al. 2022). The geographic position of Gorongosa National Park lies at the interface of the areas occupied by three species of *Papio*. The local population of chacma baboons ( *P. ursinus* ) is in fact exposed to potential interactions not only with yellow baboons ( *P. cynocephalus* ) from the north but also with kinda ( *P. kindae* ) from the west. Not surprisingly, Mozambique has been long suggested as an area of interest to investigate the population dynamics and evolutionary consequences related to hybridisation events (Jolly 1993; Burrell 2009; Martinez et al. 2019; Kopp et al. 2023). Recently, genomic analyses revealed that the Gorongosa population of baboons had experienced introgression from yellow baboons (Santander et al. 2022) and that the introgression was reflected in the great variation in morphology displayed by local animals (e.g., showing diagnostic features of yellow and chacma baboons, Martinez et al. 2019). However, it has not yet been assessed how the *P. ursinus* population living in this geographically significant area is genomically related to other *P. ursinus* populations in the region. Moreover, it remains unclear whether the hybridisation signals observed by Santander et al. (2022) are similar to those found in nearby populations involved in hybridisation events (such as the population living in Zambia; Jolly et al. 2010; Chiou et al. 2021; Sørensen et al. 2023) or if the hybridisation signals in different populations are indeed distinct, possibly because they stem from entirely different events.

At the time of the publication of the first genomic data from Mozambican baboons, *Papio* whole genomes were restricted to a handful of individuals from the wild (Wall et al. 2016; Rogers et al. 2019; Santander et al. 2022), preventing a more systematic search for the *P. cynocephalus* source population. Limitations also extended to the genomic variation sampled within *P. ursinus* . In fact, while a representative sampling of *Papio* mitogenome diversity highlighted the polyphyletic status of *P. ursinus* mitochondrial genomes (Zinner et al. 2013; Roos et al. 2021), *P. ursinus* genomic data was limited only to two Gorongosa genomes (one with very low coverage) and two captive individuals of unknown provenance (Rogers et al. 2019). These results, while highlighting the peculiarity of baboons at the northeast boundary of *P. ursinus* range, left untested their affinity to different groups of *P. cynocephalus* and to what extent introgression was present in other groups of *P. ursinus* .

Recently, an extensive survey of *Papio* genomic variation comprising more than 200 individuals has been published (Sørensen et al. 2023). This investigation highlighted multiple hybridisation events between *Papio* species; in particular, the yellow baboons from western Tanzania were described as the result of admixture between yellow, kinda, and olive baboons ( *P. anubis* ), while the yellow baboons from eastern Tanzania showed only contribution from olive baboons in their genome. Unfortunately, despite investigating hundreds of *Papio* genomes, only four *P. ursinus* samples were included in this work. Interestingly, these samples originated from Dendro Park, in Zambia, approximately 1000 km west of Gorongosa and similarly at the northern boundary of *P. ursinus* range extension. The analysis of Dendro Park *P. ursinus* revealed that this population is characterized by recent admixture with kinda and yellow baboons (< 10 generations). The larger contribution of *P. cynocephalus* present on the X chromosome than the rest of the genome was interpreted as a male‐biased admixture event. However, no test has been run yet to evaluate possible differences in signals of hybridisation of the neighboring species *P. cynocephalus* and *P. kindae* with chacma populations outside of their northern range in Zambia (Sørensen et al. 2023).

Following these observations, we decided to extend the work published by Santander et al. (2022) by leveraging the recently published genomic survey of baboon variation (Sørensen et al. 2023) to refine and further characterize the evolutionary links between baboons in Central Mozambique (Gorongosa National Park and Catapù Reserve) with neighboring *P. ursinus* populations and other *Papio* species. We combined the analysis of uniparental genetic markers (mitochondrial and Y chromosome) with the investigation of autosomal genome‐wide and X‐chromosome variants, adding unpublished low‐coverage genomic sequences from fecal samples collected in Gorongosa and Catapù Forest, and placed these data in the context of the Sørensen et al. (2023) dataset, the most complete *Papio* genomic survey to date. Since markers have not been analyzed in all the samples, we assembled three distinct datasets (autosomal, mitochondrial, Y and X chromosome), each containing the same populations (where possible) but with the inclusion of additional samples when relevant for the analyses (e.g., sampling otherwise missing diversity). Overall, our aim is to provide an updated evaluation of the relationships of Central Mozambique baboons with other baboon populations, with a particular focus on recently released genomic data of *Papio ursinus, Papio cynocephalus*, and *Papio kindae*. Implications for future work in Gorongosa, and more generally for *Papio* evolutionary history, are also discussed.

## 2 Materials and Methods

### 2.1 Samples, Sequencing Data and Comparative Datasets

**FIGURE 1:**
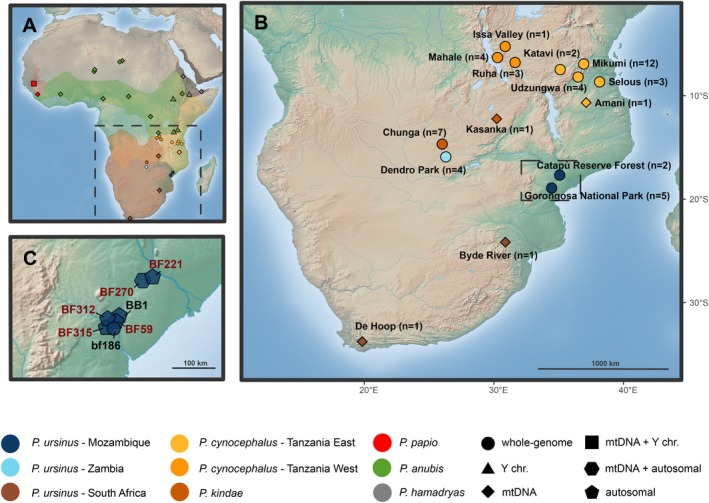
Maps of locations of analyzed baboon samples. (A) Mozambique samples origins, with newly generated genome samples highlighted in red; (B) Southern clade baboon samples localities and number of samples used per locality; (C) baboon samples localities and species ranges based on Sørensen et al. (2023). In A, B, and C the colors and shapes are referred to in the caption (see Table S1 for more details).

Fecal samples (~200) were collected in 2017–2018 in Gorongosa National Park and Catapú Reserve Forest (150 km north of the Park; Figure 1C) and preserved until DNA extraction using the “two‐step protocol” (Roeder et al. 2004). Total genomic DNA was extracted using the QIAamp DNA Stool Mini Kit (QIAGEN; see the modifications from the manufacturer's protocol in Ferreira da Silva et al. 2014). A set of 14 autosomal microsatellite loci was amplified via PCR (Ferreira da Silva et al. 2025) and used to estimate the “Quality Index” QI, (Miquel et al. 2006), a measure of the reliability of the consensus genotypes used also as a proxy of quality and quantity of nuclear DNA in non‐invasive DNA samples (Miquel et al. 2006). Six samples with mean QI > 0.9 wereselected for endogenous DNA enrichment following Chiou & Bergey's methylation‐based protocol (Chiou and Bergey 2018). This method—named FecalSeq—uses proteins in the methyl‐CpG‐binding domain to isolate DNA with high CpG‐methylation density, such as the DNA from vertebrates (Chiou and Bergey 2018). Since bacterial DNA is expected to have low CpG‐methylation densities, the bait preferentially binds to host DNA (Chiou and Bergey 2018). Enriched samples were sequenced in a single lane (PE150) by Novogene on Illumina NovaSeq 6000 S4 flowcell platform (BF270) and by Edinburgh Genomics on Illumina MiSeq platform (BF59, BF221, BF312, BF315), reaching the mean depth coverage across samples of 0.024X (0.006–0.057; Tables S1 and S2). The sexing of the fecal samples was determined by Ferreira da Silva et al. (2025). Unfortunately, only BF221 was successfully sexed, as a female.

One high‐coverage (36.8X) and one low‐coverage (0.03X) genome from Gorongosa National Park, Mozambique, already available (bf186 and BB1 in Santander et al. 2022; male and female, respectively) and the five new low‐coverage genomes (BF59, BF312, BF315 from Gorongosa National Park; BF221, BF270, from Catapù Reserve Forest; Figure 1B) were merged with a selection of genomes from Sørensen et al. (2023) (see Table S1; Figure 1C). The autosomal dataset (Figure 1; Table S1) included all the chacma baboons from Zambia plus a subset of *P. kindae* and a subset of *P. cynocephalus*, both sampled from each of the locations included in (Sørensen et al. 2023) (reported as eastern for samples collected in Mikumi, Ruha, Udzungwa, Seous; and western, for samples originating in Issa Valley, Mahale and Katavi; Figure 1B). Other *Papio* species were not included in the autosomal dataset, as the focus of this work is the characterization of the relations of Mozambican baboons with neighbor populations and species (Jolly 1993). The final dataset was composed of 56 samples, including *M. leucophaeus* as the outgroup.

For the mitochondrial dataset (Figure 1; Table S1), we extracted whenever possible mitochondrial sequences from the newly generated samples (for more information, see the Mitochondrial Analysis section) and then included all the whole mitochondrial DNA sequences from previous *Papio* mitogenomic investigations (Zinner et al. 2009; Roos et al. 2021; Figure 1A) and the mitogenomic data generated by Sørensen et al. (2023) provided by Roos, which was limited to focus on the three southern *Papio* species (*P. ursinus, P. kindae* and *P. cynocephalus* ). When identical mitogenomes were identified, only one sequence was included, as described in Table S7 of Sørensen et al. (2023). We finally added the mitogenome previously recovered from the genome of a baboon from Gorongosa Park (bf186; Santander et al. 2022). A total of 43 mitogenomes were included in the final analysis (Table S1). The mitogenome recovered from *Theropithecus gelada* (NCBI accession NC_019802.1) was used as an outgroup for mitochondrial analyses.

For the Y chromosome dataset (Figure 1; Table S1), we visually inspected the Y chromosome tree in Sørensen et al. (2023) and selected samples based on these criteria: (i) for the populations absent in the Gorongosa hybrid zone (*P. hamadryas*, northern *P. anubis*, southern *P. anubis* and *P. papio*; Figure 1A) we selected the most representative sample per species; (ii) for the southern species ( *P. kindae* , *P. ursinus* , *P. cynocephalus* ) we selected a subset of samples representing the phylogenetic diversity observed in the tree; (iii) we also included two samples that represent clear exceptions in the phylogeny to verify that the same topology was recovered: the western yellow baboon PD0231 and the southern olive baboon PD0642. The final dataset included a total of 25 samples: 24 from Sørensen et al. (2023), including one *T. gelada* sample used as an outgroup, and the previous Gorongosa high‐coverage genome bf186 (Santander et al. 2022). For each sample, the corresponding reads were downloaded from ENA (see Table S3).

Northern *Papio* species were added to the analysis of Y chromosome and mitochondrial DNA to allow comparisons with previous phylogenetic analyses.

The details of the samples included in each analysis (autosomes, mitogenomes, Y and X chromosomes) are presented in Table S1.

### 2.2 Autosomal and X Chromosome Mapping

In order to recover the autosomal variants, we initially mapped the reads to the reference genome Panubis1 (Batra et al. 2020) with *BWA‐MEM2* v.2.2.1 (Vasimuddin et al. 2019). We then marked the duplicate reads with *Picard MarkDuplicates* version 2.8.1 (http://broadinstitute.github.io/picard/) and filtered the result using the option ‐q 10, ‐F 1292, and ‐f 2 of *samtools view* (Danecek et al. 2021). We used *Mandrillus leucophaeus* (drill) as an outgroup for autosomal analyses (from NCBI project PRJNA785018). The drill genome was mapped to Panubis1.0 as indicated above. The resulting bam files for the newly generated fecal samples (BF59, BF221, BF270, BF312, BF315) were used to recover statistics through the command qualimap bamqc (García‐Alcalde et al. 2012; Table S2; Figure S1).

### 2.3 Mitochondrial Analysis

To extract the mitochondrial sequences from the genomic data, we mapped the reads to the mitochondrial *P. anubis* reference genome NC_020006.2 with *BWA‐MEM2* v.2.2.1 (Vasimuddin et al. 2019). Mitochondrial‐mapped reads were then extracted with *bcftools* v.1.19 (Danecek et al. 2021), generating a consensus *fastq*. Conversion from fastq to fasta was performed using Seqtk (https://github.com/lh3/seqtk), using default settings. Of the newly generated fecal samples, we were able to extract usable sequences (sequences with less than 85% of gaps or ambiguous bases when confronted with the reference mitochondrial sequence) mitogenomes only for one (BF270; 69.7% of gap/ambiguity). Although the ratio between nuclear and mitochondrial DNA in a given cell would technically facilitate mitochondrial DNA extraction, the general low content of endogenous DNA in fecal samples in addition to the preference for the capture extraction method to perform better at the extraction of nuclear DNA (Chiou and Bergey 2018) makes it harder to extract whole mitochondrial genomes from fecal samples, and as such it is not surprising that only one of the analyzed samples provided mitochondrial DNA sequences amenable to further analysis. The newly extracted mitogenome and mitogenomes added from previous publications (Zinner et al. 2009; Roos et al. 2021; Santander et al. 2022; Sørensen et al. 2023); (see Table S1) were rotated using *Circlator* version 1.5.5 (Hunt et al. 2015) using the—genes option and setting as a starting point the Phenylalanine tRNA sequences. All mitogenomes were aligned using MAFFT v.7.520 (Katoh and Standley 2013) with the—auto option, using NC_019802.1 ( *Theropithecus gelada* ) as an outgroup. Gap‐rich regions were trimmed using *trimAl* v.1.4.rev15 (Capella‐Gutiérrez, Silla‐Martínez, and Gabaldón 2009) with the *‐gappyout* option. Maximum Likelihood mitochondrial trees were generated with IQ‐TREE v.2.2.6 (Minh et al. 2020). ModelFinder (Kalyaanamoorthy et al. 2017) was used to infer the best model, which was determined to be TN + F + I + R3, and branch support values were determined using 1000 Ultrafast bootstraps. IQ‐TREE was also used to determine divergence times using the least‐square dating (LSD2) method and default settings (Standard Deviation of lognormal relaxed clock: 0.2) (To et al. 2016). The tree was calibrated by manually setting the root node (*Theropithecus‐Papio*) based on the oldest known *Theropithecus* fossil (4.2 Mya; (Jablonski and Frost 2010)). Confidence intervals were determined with default IQ‐TREE parameters by resampling each branch length 100 times.

### 2.4 Y Chromosome Analysis

All samples were processed with Grenepipe (Czech and Exposito‐Alonso 2022), a scalable pipeline for variant calling, with default parameters mapping to *P. anubis* (Panubis1; Batra et al. 2020) and to *Macaca mulatta* (*Mmul 1.0*). We mapped to both references to compare results obtained when different references were used as a way to evaluate the potential for reference‐driven biases. SNPs were called and filtered using the same parameters as the autosomal ones (see next section). Only biallelic and non‐heterozygous SNPs were considered for phylogenetic inference (with bcftools view—max‐alleles 2—exclude‐types indels—genotype hom, bcftools v1.19; Danecek et al. 2021). The quality of the sequences of the fecal samples was such that phylogenetically informative variants were not retrieved and therefore none of the fecal samples was included in the Y chromosome analysis (data not shown). Each Y chromosome haplotype sequence was then extracted with gatk FastaAlternateReferenceMaker v.4.5.0. All sequences were then merged and a maximum likelihood tree was generated with IQ‐TREE v.2.2.0 (Minh et al. 2020) with 1000 Ultrafast bootstrap (Hoang et al. 2018) and automatic model selection (Kalyaanamoorthy et al. 2017). The topology inferred was then dated with IQ‐TREE using the same strategy as the mitochondrial tree.

### 2.5 Autosomal and X Chromosome SNPs Analysis

#### 2.5.1 Variant Calling

We called the autosomal chromosome variants, using the GATK version 4.2.4.1 (McKenna et al. 2010). With *HaplotypeCaller* we generated the gVCF for each sample, and we made the joint calls via *GenotypeGVCFs*. The VCF thus obtained was filtered with VariantFiltration using hard filters for SNPs (“QD < 2.0 || MQ < 40.0 || FS > 60.0 || MQRankSum< −12.5 || ReadPosRankSum < −8.0”). Only for the X chromosome variants, we set ploidy 2 for males and then filtered out erroneously called heterozygous calls. All the non‐biallelic sites were removed with PLINK 1.9 (Chang et al. 2015) using the*—snps‐only* option. The final set of analyzed markers included 24,220,997 sites (autosomal and X chromosomes). We called the variants of BB1 and Mozambican fecal samples through the *pileupCaller* (https://github.com/stschiff/sequenceTools; Lamnidis et al. 2018) software for low‐coverage data using the set of SNPs described above.

#### 2.5.2 Subsampling

In order to validate the high‐coverage sample bf186 variant calling through GATK and the low‐coverage BB1 and fecal samples here generated variant calling through PileupCaller, we subsampled bf186 to a comparable coverage (0.01X) with picard DownsampleSam as in Santander et al. (2022). We then called the autosomal variants with the same low‐coverage tool PileupCaller as described above and ran the subsequent analysis.

#### 2.5.3 PCA

We performed a principal component analysis (PCA) using the *smartpca* function implemented in EIGENSOFT software 8.0.0 (Patterson et al. 2006), using *lsqproject* to project the low‐coverage samples. All the settings were used with default parameters.

#### 2.5.4 *D*‐Statistics

We tested the occurrence of imbalances in allele sharing between different populations and species (H1, H2, and H3), including drill ( *M. leucophaeus* ) as an outgroup (H4), using *qpDstat* (Patterson et al. 2012), with default parameters.

In the absence of gene flow, conflicting allelic patterns occur with equal frequency, resulting in D values of zero. When gene flow is present, there is an overrepresentation of one of the allelic patterns, leading to a deviation of D from zero. A positive D signifies introgression between H1 and H3, while a negative D indicates gene flow between H2 and H3. Observations are deemed significant if the Z‐score is greater than 3.

We initially tested to what extent the fecal Mozambican samples showed patterns of shared alleles similar to the high coverage sample, by comparing high (bf186; as H1) and low‐coverage samples (BB1, BF221, BF270, BF312, BF315, BF59; as H2), vs. all the other populations ( *P. ursinus* Zambia, *P. cynocephalus* eastern, *P. cynocephalus* western or *P*. *kinda*; alternatively included as H3). We repeated the same analysis by including as a low coverage sample the high coverage Mozambican sample (bf186) resampled to a median coverage of 0.01X (bf186_subsampled). To identify differences between *P. ursinus* in Zambia and in Mozambique in relation to other southern *Papio* species/populations, we implemented two series of *D*‐statistics analyses. In one, we explored the degree of allele sharing of the two available wild chacma populations (alternatively including as H3 either Zambia or Mozambican samples—as high coverage bf186, individual low coverage samples—including bf186_subsampled‐ or merging all the low coverage samples to generate a low coverage Mozambique population) with the populations of yellow baboons (eastern and western) and kinda (as H2 and H1, in three different combinations: yellow eastern‐yellow western; yellow eastern‐kinda; yellow western‐kinda). In the other, we compared to what extent Mozambican samples described above (as H1) and Zambia (as H2) shared alleles with *P. cynocephalus* east, *P. cynocephalus* western or *P. kinda*, alternatively included as H3.

## 3 Results

In this study we placed the genomic variation of chacma baboons from Gorongosa National Park in the context of a newly available dataset (Sørensen et al. 2023) of whole genomes from southern *Papio* populations (Figure 1). We extended the previously generated genomes from baboons in Gorongosa National Park (Santander et al. 2022) and its proximities by recovering genome sequences from a set of fecal samples of chacma baboons collected in the Park and Catapú Reserve Forest (Figure 1C; Ferreira da Silva et al. 2025).

The fecal samples generated in this study generally have low quality. Specifically, the extraction and sequencing processes described did not yield genomes with optimal characteristics. The coverage depth, for instance, is ≤ 0.05 across all samples, dropping as low as 0.006 (BF59; Table S2). Similarly, the percentage of genome covered is also very low, ranging from 0.6% to 5.6% (Table S2). The coverage distribution across the autosomes is highly uneven, with most chromosomes showing very high peaks toward their ends (Figure S1; Table S2). Additionally, chromosome 20 consistently exhibits the highest coverage compared to all the others (Table S2). The percentage of mapped reads is relatively low as well, ranging between 16% and 52%, while the percentage of called variants is extremely low, at only 0.18% to 1.35% (Table S2).

### 3.1 Uniparental Markers Analysis

**FIGURE 2:**
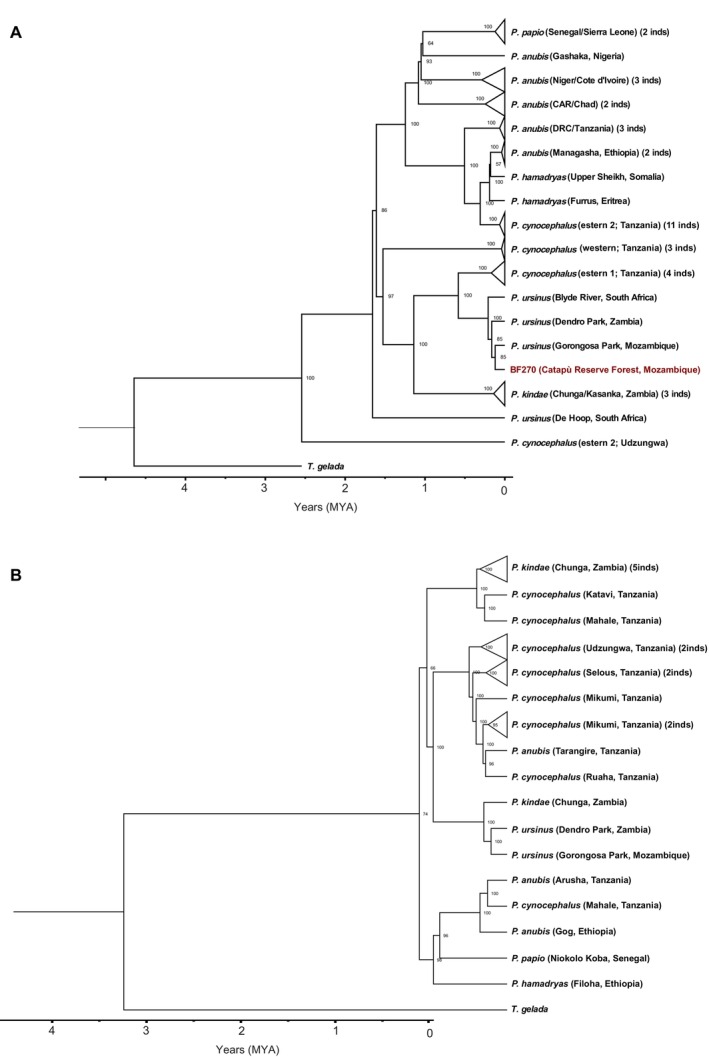
Phylogenetic analysis of uniparental systems. (A) Mitochondrial DNA tree based on 43 mitogenomes. For *P. cynocephalus* , eastern 1 clade comprises baboons from Selous and Amani, eastern 2 includes baboons from Mikumi, Udzungwa, and Ruaha, and western refers to baboons from Mahale and Issa Valley. For correspondence with clades reported in (Roos et al. 2021; Sørensen et al. 2023), see Table S5. (B) Y chromosome tree based on 25 samples. For *P. cynocephalus*, eastern and western locations as in Figure 1. Colors as in Figure 1. Labels are ordered as follows: Species (P: *Papio*; T: *Theropithecus*), sampling location (whenever available), and country of origin (CAR: Central African Republic; DRC: Democratic Republic of Congo). For collapsed clades, the number of individuals is also reported. Uncollapsed trees with accession IDs for each sample and node TMRCAs with confidence intervals are available in Supporting Information (Figure S2, Tables S4 and S6). Bootstrap values over 1000 resamplings are reported near nodes.

We analyzed the current mitochondrial diversity of *P. ursinus* in Mozambique, assembling a dataset of *Papio* mitogenomes and characterizing the phylogenetic relationship between our newly generated partial mitogenome from fecal material (BF270) and selected *Papio* samples from several previous works (Figure 2A; Figure S2A; Table S1). Overall, the tree topology is in concordance with what was presented by both Roos et al. (2021) and Sørensen et al. (2023). *P. ursinus* samples are divided between southern and northern samples, with northern samples forming a clade with *P. cynocephalus* samples from Tanzania and *P. kindae* samples from Zambia. Our newly generated sample, BF270, clusters with this northern clade, closely related to the previously published Gorongosa *P. ursinus* mitogenome (bf186; (Santander et al. 2022)), with a TMRCA dated to 120 kya (Figure 2A; Figure S2A; see Table S4). Both of these samples form a clade sister to *P. ursinus* samples from Dendro Park, Zambia (Sørensen et al. 2023). In order to understand whether the differences between bf186 and BF270 were due to the high number of ambiguous bases and the overall poor quality of the fecal samples or signified a real mitogenomic diversity inside of Central Mozambique, we tested our newly generated sample for sequence similarity with the other Gorongosa park sequence (bf186), only taking into account positions with unambiguous bases (for a total of 5087 bases considered), yielding a similarity of 99.6%. For context, when comparing the similarity of bf186 and BF270 with the closest *P. ursinus* sequences (the mitogenomes from Dendro Park, Zambia), the similarity was 99.5%. The Time to the Most Recent Common Ancestor (TMRCA) between bf186 and BF270 was dated 0.12 Mya (120 kya; confidence interval: 0.09–0.16 Mya; Table S4). The TMRCA between the central Mozambican clade and the Zambia *P. ursinus* mitogenome is dated at 0.16 Mya (0.13–0.22 Mya; Table S4) while the divergence between the *P. ursinus* from northern South Africa (Blyde River, South Africa) and the Zambia/Central Mozambique clade is dated at 0.21 Mya (0.15–0.29 Mya; Table S4). The divergence between all northern *P. ursinus* samples and eastern *P. cynocephalus* (identified as “Clade B” in Sørensen et al. (2023)) is dated at 0.58 Mya (0.43–0.78 Mya; Table S4), while the TMRCA of the clade containing all *P. ursinus* lineages, northern and southern, is dated at 1.65 Mya (1.4–1.93 Mya; Table S4). These divergence times, when directly comparable, are consistent with previous estimates: for instance, the divergence between northern *P. ursinus* samples and the eastern Clade B *P. cynocephalus* was dated at 0.74 Mya (CI: 0.55–0.94 Mya) by Zinner et al. (2013), 0.82 Mya (CI: 0.61–1.02 Mya) by Roos et al. (2021), 0.55 Mya (0.38–0.73 Mya) by Santander et al. (2022), and 0.57 Mya by Sørensen et al. (2023) (Table S4).

For the Y chromosome, we assembled a dataset with sequences from Sørensen et al. (2023) to cover most of the range of *Papio* species. Bf186 groups together with maximum support with the only other *P. ursinus* sample in the tree, an individual from Dendro Park in Zambia with a TMRCA dated to 170 Kya (CI: 260–130 Kya). The next node links *P. ursinus* to one of the two *P. kindae* lineages represented in the phylogeny, with a coalescent time very close to the one between the two *P. ursinus* lineages (Figure 2B; Figure S2B; Table S6). It is worth noticing here that the topology of this uniparental marker presents some differences to the one presented in Sørensen et al. (2023). Firstly the position of the *P. kindae* ‐western *P. cynocephalus* clade, which is sister group to all other *Papio* in Sørensen et al. (2023), in our phylogeny falls inside of a clade shared with other southern *Papio*. This difference in the topology could be caused by the use of Panubis1 as a reference genome for mapping. Indeed, we retrieve the same topology as Sørensen et al. when mapping the samples to the *Macaca mulatta* reference genome (Mmul 1.0) as they did (Figure S3; Table S6). Another difference in the Y chromosome phylogeny generated by mapping to *P. anubis* is that estimated dates are more recent for nodes with the same topology as Sørensen et al. (2023)'s compared to those estimated by Sørensen et al. (2023). In contrast, in the phylogeny generated by mapping to *M. mulatta* , the dates are older for the more recent nodes, while they align with those identified by Sørensen et al. (2023) for the older nodes (Table S6). However, even if more recent, the coalescent time between *P. kindae* and the two *P. ursinus* overlap with that of the two *P. ursinus* , as for the Panubis1‐based tree.

### 3.2 Autosomal and X Chromosome Analysis

**FIGURE 3:**
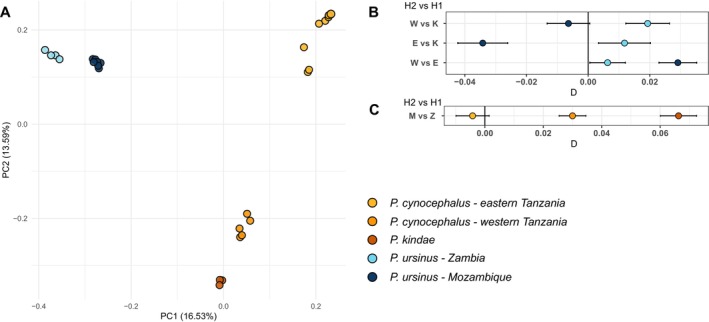
Analysis of autosomal variants (A) PCA of autosomal genome‐wide markers; (B) *D*‐statistics comparing neighboring species ( *P. cynocephalus* from eastern Tanzania, *P. cynocephalus* from western Tanzania and *P. kindae* as H2 and H1) to chacma populations (Mozambique and Zambia as H3). (C) *D*‐statistics comparing chacma populations (same as B; as H2 and H1) to neighboring species (same as B; as H3). In B and C, the bars show the extension of three standard deviations and the colors refer to the taxon used as H3 as indicated in the legend; In B and C, the H1 and H2 labels refer to *P. cynocephalus* from eastern (E) Tanzania, *P. cynocephalus* from western Tanzania (W), *P. kindae* (K), *P. ursinus* from Zambia (Z) and *P. ursinus* from Mozambique (M). For positive values, the signal of gene‐flow is between H3 and H1, while for negative values the signal is between H3 and H2.

The PCA of autosomal genome‐wide markers highlighted three main clusters, mostly reflecting the three species (Figure 3A). The main exception was *P. cynocephalus* baboons from western Tanzania (Issa Valley, Mahale, Katavi), which appeared closer to *P. kindae* than *P. cynocephalus* from eastern Tanzania (Mikumi, Ruha, Udzungwa, Selous), as previously reported (Sørensen et al. 2023). *P. ursinus* populations from Mozambique and Zambia clustered close to each other. The low‐coverage genomic samples recovered from fecal material formed a cloud around the high‐coverage bf186 and the low‐coverage BB1 samples previously published, suggesting a general genomic similarity for samples from Gorongosa National Park and Catapù Reserve Forest.

We then tested, through *D*‐statistics, whether individuals with low coverage and the individual with high coverage (bf186) from Mozambique were equally related to other species and to the chacma population in Zambia. We observed that overall, with some degree of interindividual variation, samples tend to behave similarly in relation to other species and populations (Figure S4A). For this reason, we decided to consider all individuals from Gorongosa National Park and Catapù Reserve Forest as a single population in subsequent analyses. The patterns of allele sharing of the two populations of *P. ursinus* with the two groups of *P. cynocephalus* highlighted a broad similarity between the chacma groups (Figure 3B). Both share more alleles with eastern than western yellow baboons (*D* = 0.0063 *Z* = 3.293, for Zambia; *D* = 0.0291 *Z* = 14.257, for Mozambique). However, when we compared the two populations with *P. kindae* the pattern was different, the Zambian population shared more alleles with this species than the Mozambican one (*D* = 0.0663 *Z* = 32.130). Similarly, *P. ursinus* in Zambia was closer to western yellow baboons than *P. ursinus* in Mozambique (*D* = 0.0300 *Z* = 19.645) (Figure 3C). The same results were observed when only the high‐coverage Gorongosa sample (bf186) was included in the analyses (Figure S4B,C). However, some degree of interindividual variation was observed.

As such, we rerun the analysis of autosomal data (PCA and *D*‐statistics; Figure S5) including the bf186 specimen subsampled at a coverage of 0.01X (Table S2), calling variants together with other low coverage samples (BB1 and the other fecal samples generated here) using PileupCaller. The high coverage bf186 sample and the subsampled bf186 behave in a similar way when compared to other populations but differ at times when compared to each other, or when they are compared to Zambia (Figure S5A–D). However, we noted that the subsampled bf186, contrary to the high coverage genome sample bf186, does not differ from other low coverage samples (Figure S5E). Taken together, these two observations suggest that interindividual differences might be driven by limited data in the low coverage samples. We tested this hypothesis by merging all of the low coverage samples to generate a low coverage Mozambican population and compared it to the high coverage sample (bf186). The results are similar to those obtained for the subsampled bf186 when compared to the high coverage (Figure S5C,D,F,G). However, differently from the single low coverage samples (Figure S4), the low coverage Mozambican population behave similarly to the high coverage sample bf186 when they are considered as H3 and other *Papio* species/populations are included as H1 and H2 (Figure S5F).

We also ran PCA on X chromosome markers, which showed a tripartition of the three species, as observed with the autosomal markers (Figure S6A). We also noted that the fecal samples are the most shifted toward the origin of the coordinates, particularly the two with the lowest number of called variants (bf312 and bf59; Table S2), while the others are located near bf186.

We then tested the allele‐sharing patterns of the two *P. ursinus* populations (Zambia and Mozambique as H3; Figure S6B) via *D*‐statistics using X chromosome variants. No significant difference between *P. cynocephalus* from eastern Tanzania, western Tanzania, and *P. kindae* was observed in their affinity for the two *P. ursinus* populations. Conversely, when we tested the reverse scenario (with *P. kindae* , *P. cynocephalus* from eastern or western Tanzania alternately as H3, and Zambia and Mozambique as H1‐H2; Figure S6C), we observed a pattern similar to that of the autosomal markers: there was higher allele sharing with the population from Zambia compared to the one in Mozambique, although the intensity was lower.

## 4 Discussion

Mitochondrial DNA analysis highlighted some degree of variation within Mozambique, the two mitogenomes recovered so far seemingly presenting some differences and a relatively recent TMRCA (120 kya). Notably, the mitogenomes of bf186 (sampled in the core area of Gorongosa National Park) and BF270 (sampled in Catapù Forest Reserve, approximately 150 km apart) are 99.6% similar, and just slightly less so at 99.5% with *P. ursinus* mitogenomes from Dendro Park in Zambia. The low mitogenome diversity in Mozambique is in line with the reported limited mitochondrial variation observed locally in a larger sample of mtDNA fragments reported elsewhere (Ferreira da Silva et al. 2025). More variation is expected in the region as suggested by the retrieval of a divergent haplotype in the nearby Catapù area (Ferreira da Silva et al. 2025) where BF270 was also sampled. Additional high‐quality mitochondrial genomes from Gorongosa Park are therefore essential to provide a finer description of local mtDNA variation. The clustering of Central Mozambique mitogenomes with Zambia *P. ursinus* rather than the *P. ursinus* sample from Blyde River in northern South Africa suggests a closer affinity of these locations, possibly reflecting a north–south cline inside the northern *P. ursinus* range. The estimated TMRCAs for the northern *P. ursinus* clades at 0.58 Mya date to a period of climate‐driven habitat fragmentation that characterized the late Pleistocene (Sithaldeen et al. 2009).

Contrary to mtDNA, Y chromosome variation has been surveyed minimally in *Papio*, even more so for *P. ursinus*, the only data available being the one from Zambia and Mozambique here discussed. More data is necessary to properly investigate the phylogenetic relationships of male lineages, within and between populations (Mutti et al. 2023). Overall, divergence times of uniparental markers of the northern *P. ursinus* specimens tell a similar tale: the split between the Zambia *P. ursinus* and the Gorongosa park clade is dated around 0.16 Mya for the mitochondrial dataset (CI: 0.13–0.22 Mya) and at 0.17 Mya for the Y chromosome (CI: 0.13–0.26 Mya) markers. The closest species for this group of *P. ursinus* is a *P. kindae* sample for the Y chromosome and *P. cynocephalus* from eastern Tanzania for the mitochondrial DNA. However, it is interesting to note that the coalescent time with *P. kindae* for the Y chromosome is similar to the TMRCA between Zambia and Mozambique (0.25 Mya vs. 0.17 Mya), especially when taking into consideration the confidence intervals of these splits (0.38–0.19 Mya and 0.26–0.13 Mya). This raises the possibility that this kindae lineage might be the result of male introgression from *P. ursinus*, even if kindae‐to‐chacma male‐mediated gene flow has been suggested in Zambia (Chiou et al. 2021; Sørensen et al. 2023). This would be one possible explanation compatible with the polyphyletic status of *P. kindae* in the tree and the deeper coalescent time of the other Y chromosome kindae lineage with *P. cynocephalus* (0.33 Mya). We finally note that while the TMRCAs differ when using Panubis1 or MMul1.0, the overlap in TMRCAs for the kindae‐chacma node and the *P. ursinus* Zambia‐ *P. ursinus* Mozambique is present in both phylogenetic analyses. However, it is clear that there is still much unsampled diversity that it is not possible to answer the question of male *P. ursinus* introgression in *P. kindae* properly. Interestingly, the next Y chromosome node of this clade is shared with *P. cynocephalus* from eastern Tanzania, as for the mitochondrial DNA, with overlapping TMRCAs (0.66–1.05 Mya for the Y chromosome and 0.43–0.78 Mya for the mitochondrial DNA).

The patterns of shared autosomal alleles highlighted by the *D*‐statistics suggest that both Zambia and Gorongosa have been exposed to *P. cynocephalus* introgression, the source possibly being a population closer to the eastern than western *P. cynocephalus* here tested. However, Zambian *P. ursinus* appear to host an additional component, related to *P. kindae* as previously suggested (Sørensen et al. 2023). A similar three‐way admixture has been suggested also for western *P. cynocephalus* (Sørensen et al. 2023) which would explain the indication of this population being closer to Zambian than Mozambican baboons. The similar affinity with eastern yellow and the differential association with *P. kindae* suggest northern *P. ursinus* populations might have been characterized by different dynamics in their interaction with nearby *Papio* species. It is important to stress here that at this stage the data is simply indicating that Central Mozambique baboons are less related to kinda or kinda‐admixed yellow baboons than Zambia chacma. This does not exclude the possibility that similar signatures are present in Gorongosa and Catapù too but, if so, these would be less relevant than those in Dendro Park baboons in Zambia. Similarly, the timing of *P. cynocephalus* contribution to Zambian and Mozambican *P. ursinus* is still to be determined, but it might not be too recent (Santander et al. 2022).

Regarding the X chromosome, in agreement with previously reported results by Santander et al. (2022) no differences in allele sharing were detected across *P. cynocephalus* and *P. kindae* with Mozambican *P. ursinus* , while a pronounced allelic affinity is observed between *P. cynocephalus* from western Tanzania and *P. ursinus* from Zambia, as opposed to those from Mozambique. The mismatch between X chromosome and autosomal results for Mozambican *P. ursinus* may imply a predominantly male introgression and/or extensive purifying selection on the X chromosome.

Mozambique, in the south‐eastern part of Africa, is located at the interface of the ranges occupied by three species of *Papio*. Naturally expected to be a hybridisation hotspot, genomic data confirmed this being the case in Mozambique. The non‐recent signatures of introgression detected in Gorongosa baboons possibly reflect the efficient barrier to gene‐flow from the north represented by the Zambezi River and the role played by other populations of *P. ursinus* in the west as stepping stones for kinda introgression (Martinez et al. 2019; Santander et al. 2022). However, we don't know anything, besides a handful of mitochondrial fragments, of the genomic status of other Mozambican populations, in particular in the north where *P. cynocephalus* is reported and in the west where populations are the closest to *P. kindae* (www.iucnredlist.org). A more extensive survey of *Papio* in Mozambique is therefore necessary to investigate more in detail the variation present in baboons in this part of Africa.

The sampling of the genomic diversity of *P. ursinus* is currently limited to a few genomes from two locations at the northernmost edges of the habitat occupied by this species. The two populations sampled in the wild belong to the subspecies *P. u. griseipes*, one of the subspecies proposed for *P. ursinus* (Jolly 1993). It is self‐evident that in order to understand the evolutionary history of this species, it is critical to expand the sampling of other populations across southern Africa. A more exhaustive sampling would allow exploring the extent of the introgression of *P. cynocephalus* and *P. kindae* , the degree of population structure, and the evolutionary history of chacma subspecies.

Despite the extremely low coverage obtained from fecal samples limiting our ability to make extensive inferences from uniparental markers, the autosomal data was in line with the signals observed using the single high coverage genome available from Central Mozambique (Santander et al. 2022). We confirmed the validity of the findings in Gorongosa National Park by expanding the dataset to include individuals from a second area of Central Mozambique, the Catapù Reserve Forest. Despite the low and uneven coverage and depth of these genomes derived from fecal samples, we were able to obtain a reasonable number of variants using a specific tool designed for ancient DNA (PileupCaller; Lamnidis et al. 2018), which accounts for regions with pseudohaploid and/or missing data. Comparisons between low coverage and high coverage samples showed some degree of variation. However, the observation that in a few instances the subsampled bf186 behaved differently from the high coverage bf186 suggests that interindividual variation might be due to limited genomic information available for the low coverage samples. The same is also the case when multiple low coverage samples were merged, but not in all cases. In particular, the low coverage population presents the same results as the high coverage sample when used as H3 in the *D*‐statistics test, an observation that would be useful for future investigations focusing on the genomic analysis of non‐invasive samples. Overall, these results confirm that, properly acknowledging their intrinsic limitations, non‐invasive sampling can offer the opportunity to expand the sampling of genomic diversity in wild populations (Chiou et al. 2021).

We also note here that the newly added genomic samples from Gorongosa Park are consistent with previous results in showing a signature of introgression from *P. cynocephalus* (Santander et al. 2022), confirming this is a defining feature of the local population. Considering the lack/minimal presence of population structure within the Park and across nearby regions (e.g., Catapù; Ferreira da Silva et al. 2025), the sampling of more genomes is expected to provide the context for understanding the evolutionary dynamics that have shaped the genome‐wide distribution of these signatures of introgression and characterize their phenotypic consequences, including adaptations to the highly heterogeneous Gorongosa ecosystem (Bobe et al. 2020). In addition to exploring autosome and X chromosome variation, investigations analyzing several samples within a population have the potential to be very informative for a deeper characterization of uniparental markers as larger samples increase the probability of sampling different lineages (Y chromosome and mitochondrial DNA; Mutti et al. 2023; Ferreira da Silva et al. 2025), as these are more exposed to drift and sex‐specific population dynamics. Finally, we note that Gorongosa hosts other species of primates (Beardmore‐Herd et al. 2025; Gaynor et al. 2021), whose genomic and genetic analysis is expected to clarify to what extent Central Mozambique operated over time as a crossroads for interactions with nearby populations and species.

## Author Contributions

**Matteo Caldon:** conceptualization (equal), formal analysis (equal), writing – original draft (equal), writing – review and editing (equal). **Giacomo Mercuri:** conceptualization (equal), formal analysis (equal), writing – original draft (equal), writing – review and editing (equal). **Giacomo Mutti:** conceptualization (equal), formal analysis (equal), writing – original draft (equal), writing – review and editing (equal). **Maria Joana Ferreira da Silva:** conceptualization (equal), investigation (equal), methodology (equal), writing – review and editing (equal). **Felipe I. Martinez:** conceptualization (equal), data curation (equal), funding acquisition (equal), resources (equal), writing – review and editing (equal). **Cristian Capelli:** conceptualization (equal), supervision (equal), writing – original draft (equal), writing – review and editing (equal).

## Conflicts of Interest

The authors declare no conflicts of interest.

## Supporting information


**Figure S1.** Coverage across a reference in the autosomal chromosomes for the newly generated fecal samples BF59, BF221, BF270, BF312, and BF315. Plots are generated with qualimap bamqc as described in the Material and Methods section. Each panel refers to one of the five samples as indicated on the top. The vertical lines define the length of the chromosomes. The names of the chromosomes are derived from the NCBI RefSeq nomenclature: NC_044976.1 (chromosome 1), NC_044977.1 (chromosome 2), NC_044978.1 (chromosome 3), NC_044979.1 (chromosome 4), NC_044978.1 (chromosome 5), NC_044981.1 (chromosome 6), NC_044982.1 (chromosome 7), NC_044983.1 (chromosome 8), NC_044984.1 (chromosome 9), NC_044985.1 (chromosome 10), NC_044986.1 (chromosome 11), NC_044987.1 (chromosome 12), NC_044989.1 (chromosome 13), NC_044990.1 (chromosome 14), NC_044990.1 (chromosome 15), NC_044991.1 (chromosome 16), NC_044992.1 (chromosome 17), NC_044993.1 (chromosome 18), NC_044994.1 (chromosome 19), NC_044995.1 (chromosome 20). The figure starts on the previous page.
**Figure S2.** Uncollapsed phylogenetic trees using uniparental markers. (A) mitochondrial DNA phylogenetic tree based on 43 mitogenomes, on which Figure 2A is based. (B) Y chromosome DNA phylogenetic tree based on 25 samples, on which Figure 2B is based. Labels are ordered as follows: sequence/individual ID, species, sampling location (whenever available).
**Figure S3.** Y chromosome tree based on 25 samples mapped on *M. mulatta* reference genome. For *P. cynocephalus* , eastern and western locations as in Figure 1. Labels are ordered as follows: species (P: *Papio*; T: Theropithecus), sampling location (whenever available), and country of origin (CAR: Central African Republic; DRC: Democratic Republic of Congo). For collapsed clades, the number of individuals is also reported. Bootstrap values over 1000 resamplings are reported near nodes.
**Figure S4.** (A) *D*‐statistics comparing the high coverage (bf186 as H1) and the low coverage (the newly generated BF59, BF221, BF270, BF312, BF315 and the published BB1 as H2) individuals from Gorongosa National Park to neighboring taxa ( *P. cynocephalus* from eastern Tanzania, *P. cynocephalus* from western Tanzania, *P. kindae* and *P. ursinus* from Zambia; H3). *D*‐statistics calculated using only the high‐coverage sample (bf186) for the Mozambican population (B) comparing neighboring species ( *P. cynocephalus* from eastern Tanzania, *P. cynocephalus* from western Tanzania and *P. kindae* as H2 and H1) to chacma (Mozambique samples and Zambia population as H3); (C) comparing chacma populations (same as B; as H2 and H1) to neighboring species (same as B; as H3). In A, B and C the bars show the extension of three standard deviations and the colors refer to the taxon used as H3 as indicated in the legend; in B and C the H1 and H2 labels refer to *P. cynocephalus* from eastern (E) Tanzania, *P. cynocephalus* from western Tanzania (W), *P. kindae* (K), and *P. ursinus* from Zambia (Z). For positive values, the signal of gene‐flow is between H3 and H1, while for negative values the signal is between H3 and H2.
**Figure S5.** bf186 subsampled (A) PCA of autosomal genome‐wide markers, bf186 subsampled is highlighted; (B) *D*‐statistics comparing the subsampled high coverage (bf186_S as H2) and the high coverage sample (bf186 as H1) from Gorongosa National Park to neighboring species ( *P. cynocephalus* from eastern Tanzania, *P. cynocephalus* from western Tanzania, *P. kindae* and *P. ursinus* from Zambia as H3); (C) comparing neighboring species (same as B; as H2 and H1) to chacma bf186 subsampled (and the full‐coverage sample) as H3; (D) comparing bf186 subsampled and bf186 full‐coverage (as H2) and Zambia (as H1) to neighboring species (same as B; as H3); (E) *D*‐statistics comparing the subsampled high coverage (bf186_S as H1) and the low coverage (BF59, BF221, BF270, BF312, BF315, BB1 as H2) individuals from Gorongosa National Park to neighboring species ( *P. cynocephalus* from eastern Tanzania, *P. cynocephalus* from western Tanzania, *P. kindae* and *P. ursinus* from Zambia as H3); (F) comparing neighboring species (same as B; as H2 and H1) to Mozambique chacma low‐coverage as population (and the high‐coverage sample) as H3; (G) comparing Mozambique lowcoverage as population (Moz_lc) and bf186 (as H2) and Zambia (as H1) to neighboring species (same as B; as H3). In B, C, D, E, F and G the bars show the extension of three standard deviations and the colors refer to the taxon used as H3 as indicated in the legend; in C and D the H1 and H2 labels refer to *P. cynocephalus* from eastern (E) Tanzania, *P. cynocephalus* from western Tanzania (W), *P. kindae* (K), and *P. ursinus* from Zambia (Z). For positive values, the signal of gene‐flow is between H3 and H1, while for negative values the signal is between H3 and H2.
**Figure S6.** Analysis of X‐chromosome variants (A) PCA of X‐chromosome markers; (B) *D*‐statistics comparing neighboring species ( *P. cynocephalus* from eastern Tanzania, *P. cynocephalus* from western Tanzania and *P. kindae* as H2 and H1) to chacma populations (Mozambique and Zambia as H3). (C) *D*‐statistics comparing chacma populations (same as B; as H2 and H1) to neighboring species (same as B; as H3). In B and C, the bars show the extension of three standard deviations and the colors refer to the taxon used as H3 as indicated in the legend; In B and C, the H1 and H2 labels refer to *P. cynocephalus* from eastern (E) Tanzania, *P. cynocephalus* from western Tanzania (W), *P. kindae* (K), *P. ursinus* from Zambia (Z) and *P. ursinus* from Mozambique (M). For positive values, the signal of gene‐flow is between H3 and H1, while for negative values the signal is between H3 and H2.
**Table S1.** Dataset. Information regarding the samples used in this study, including sample name, genus and species, sampling location and country, dataset in which the sample was used (autosomal chromosomes, mitochondrial DNA, Y chromosome), and original project.
**Table S2.** Fecal samples. Information regarding the fecal samples generated in this study and the subsampling of bf186, including coverage depth, percentage of genome sequenced, GC content percentage, number of reads, percentage of mapped reads, percentage of autosome and X chromosome variants called, and non‐missing calls for autosome and X chromosome variants.
**Table S3.** Y chromosome runs. ENA codes and number of runs for each sample in Y chromosome dataset.
**Table S4.** Mitochondrial divergence times. Divergence times between mitochondrial clades in millions of years ago (Mya) with confidence intervals. Divergence times of clades are compared, whenever possible, with previously published data from Zinner et al. (2013), Roos et al. (2020), Santander et al. (2022) and Sørensen et al. (2023).
**Table S5.** Mitochondrial clades. Comparison between Mitochondrial clades identified in this work and previously established Mitochondrial clades, in particular those identified by Roos et al. (2020) and Sørensen et al. (2023).
**Table S6.** Y chromosomes divergence times. Divergence Times between Y chromosome clades in millions of years ago (Mya) with confidence intervals, for data mapped on *P. anubis* and *M. mulatta* reference genomes. Divergence times of clades are compared, whenever possible, with previously published data from Santander et al. (2022) and Sørensen et al. (2023).

## Data Availability

The genomic data of the samples here generated (BF59, BF221, BF270, BF312, BF315) are available under the ENA accession study number PRJEB74661.
