# Supplementary material for: Baboons at a Crossroads: Hybridisation Events and Genomic Links of Central Mozambique's Baboons With Papio Neighbors
Source: Am J Biol Anthropol. 2025 Jun 28;187(3):e70082. doi: 10.1002/ajpa.70082 (PMC12205352; doi:10.1002/ajpa.70082)
Supplement: Supplementary file 1 — Figure S1. Coverage across a reference in the autosomal chromosomes for the newly generated fecal samples BF59, BF221, BF270, BF312, and BF315. Plots are generated with qualimap bamqc as described in the Material and Methods section. Each panel refers to one of the five samples as indicated on the top. The vertical lines define the length of the chromosomes. The names of the chromosomes are derived from the NCBI RefSeq nomenclature: NC_044976.1 (chromosome 1), NC_044977.1 (chromosome 2), NC_044978.1 (chromosome 3), NC_044979.1 (chromosome 4), NC_044978.1 (chromosome 5), NC_044981.1 (chromosome 6), NC_044982.1 (chromosome 7), NC_044983.1 (chromosome 8), NC_044984.1 (chromosome 9), NC_044985.1 (chromosome 10), NC_044986.1 (chromosome 11), NC_044987.1 (chromosome 12), NC_044989.1 (chromosome 13), NC_044990.1 (chromosome 14), NC_044990.1 (chromosome 15), NC_044991.1 (chromosome 16), NC_044992.1 (chromosome 17), NC_044993.1 (chromosome 18), NC_044994.1 (chromosome 19), NC_044995.1 (chromosome 20). The figure starts on the previous page. Figure S2. Uncollapsed phylogenetic trees using uniparental markers. (A) mitochondrial DNA phylogenetic tree based on 43 mitogenomes, on which Figure 2A is based. (B) Y chromosome DNA phylogenetic tree based on 25 samples, on which Figure 2B is based. Labels are ordered as follows: sequence/individual ID, species, sampling location (whenever available). Figure S3. Y chromosome tree based on 25 samples mapped on M. mulatta reference genome. For P. cynocephalus , eastern and western locations as in Figure 1. Labels are ordered as follows: species (P: Papio; T: Theropithecus), sampling location (whenever available), and country of origin (CAR: Central African Republic; DRC: Democratic Republic of Congo). For collapsed clades, the number of individuals is also reported. Bootstrap values over 1000 resamplings are reported near nodes. Figure S4. (A) D‐statistics comparing the high coverage (bf186 as H1) and the low coverage (the newly generated [file AJPA-187-e70082-s001.pdf]

Supplementary Materials for

Baboons at a crossroads: hybridisation events and genomic links of central Mozambique's baboons with Papio neighbours

Matteo Caldon[1], Giacomo Mercuri[1], Giacomo Mutti[2,3], Maria Joana Ferreira da Silva[4,5,6], Felipe I. Martinez[7], Cristian Capelli[1,*]

1. Department of Chemistry, Life Sciences and Environmental Sustainability, University of Parma, Parco Area delle Scienze 11/a, 43124, Parma, Italy
2. Barcelona Supercomputing Centre (BSC-CNS), Plaça Eusebi Güell, 1-3, 08034, Barcelona, Spain
3. Institute for Research in Biomedicine (IRB Barcelona), The Barcelona Institute of Science and Technology, Baldiri Reixac, 10, 08028, Barcelona, Spain
4. BIOPOLIS Program in Genomics, Biodiversity and Land Planning, CIBIO, Campus de Vairão, Vairão, Portugal
5. CIBIO, Centro de Investigação em Biodiversidade e Recursos Genéticos, InBIO Laboratório Associado, Campus de Vairão, Universidade do Porto, Vairão, Portugal
6. ONE - Organisms and Environment Group, School of Biosciences, Cardiff University, Sir Martin Evans Building, Cardiff, UK
7. Escuela de Antropología, Facultad de Ciencias Sociales, Pontificia Universidad Católica de Chile, Santiago, Chile

*Corresponding author. Email: cristian.capelli@unipr.it

This PDF file includes:

- **Supplementary Figure 1**
- **Supplementary Figure 2**
- **Supplementary Figure 3**
- **Supplementary Figure 4**
- **Supplementary Figure 5**
- **Supplementary Figure 6**

BF_59

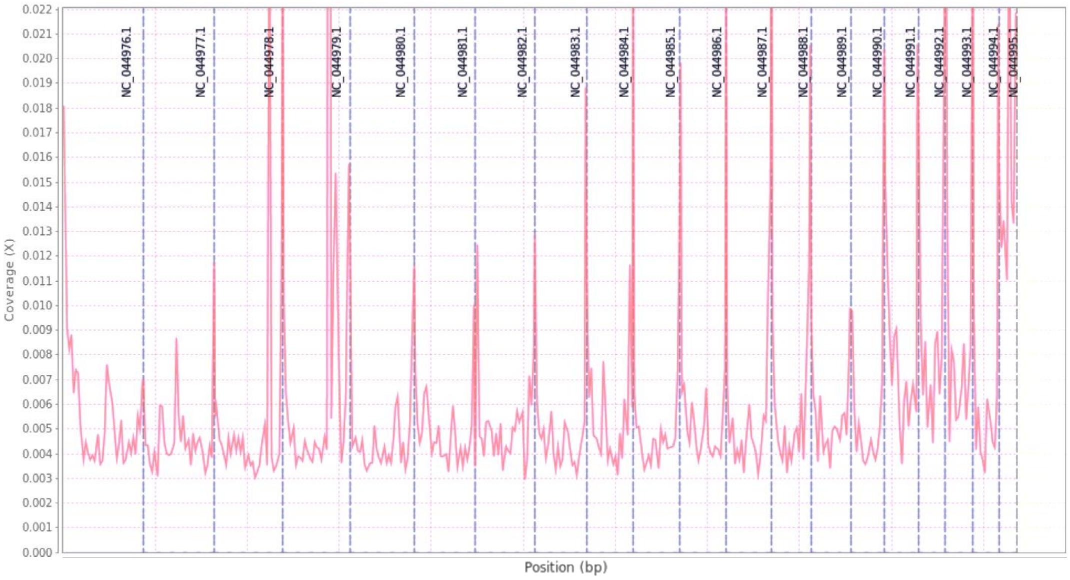

BF_221

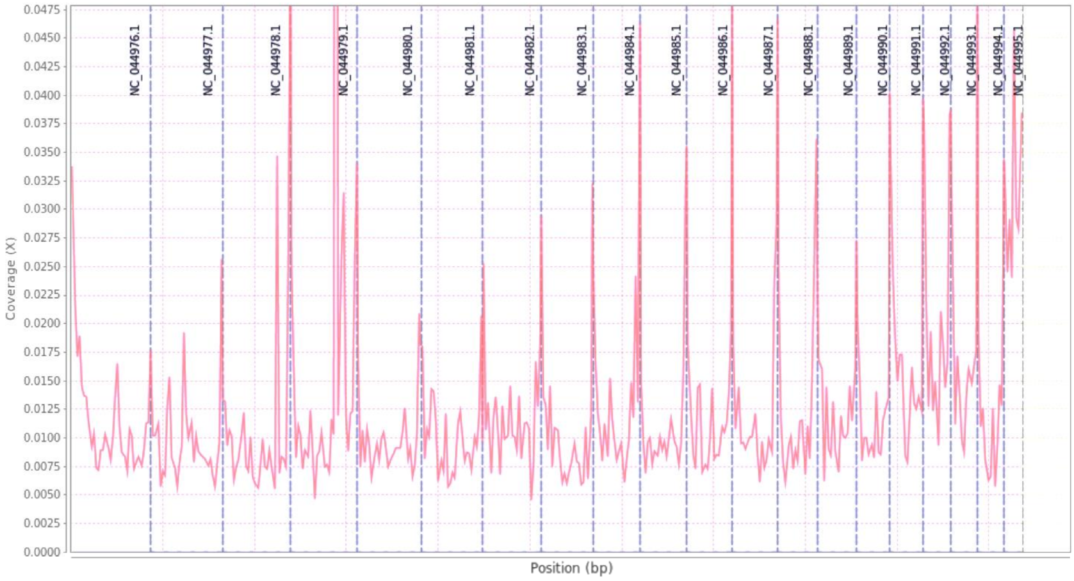

BF_270

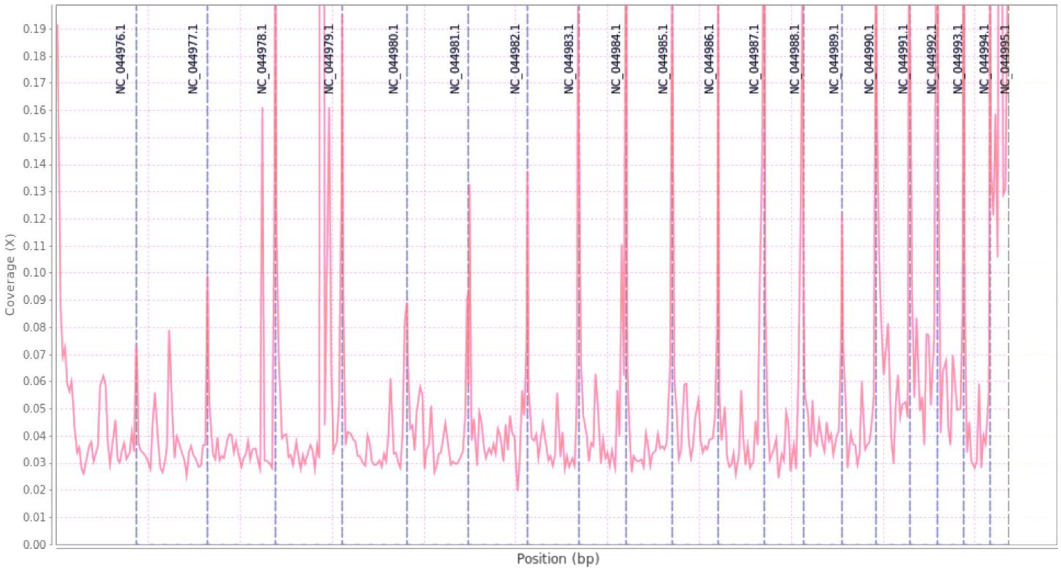

BF_312

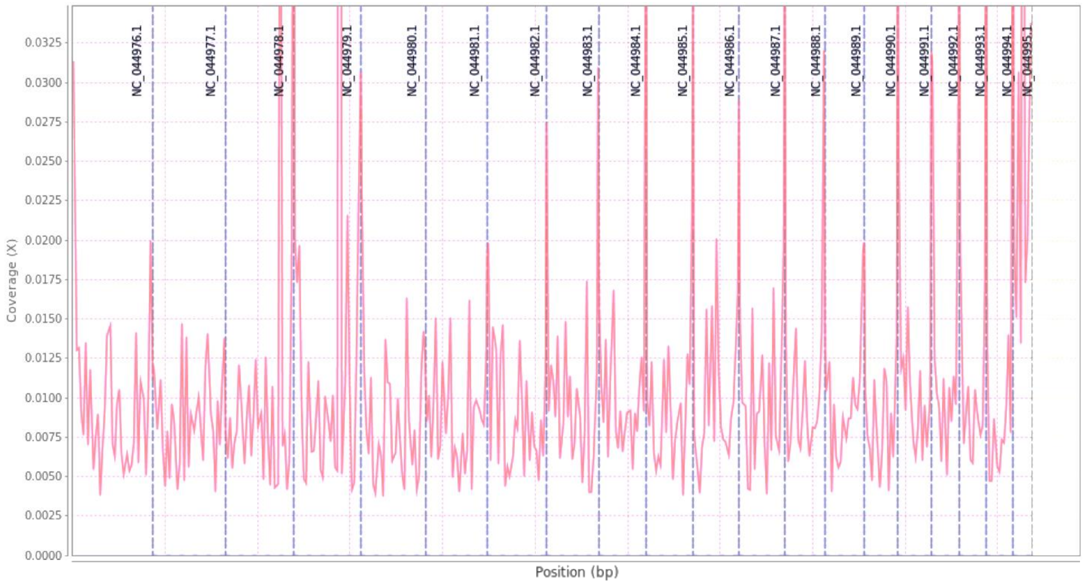

BF_315

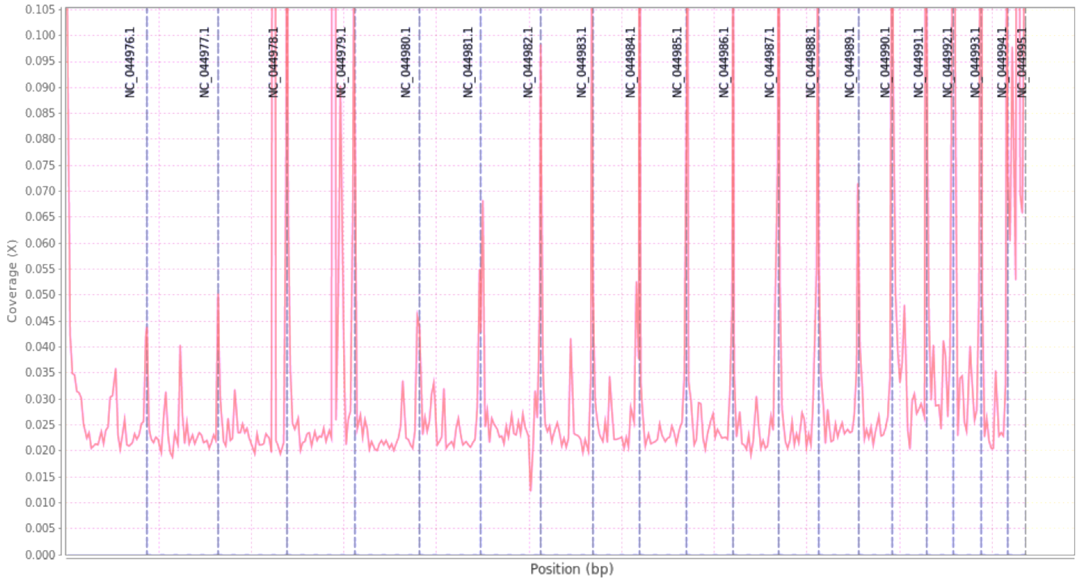

SFigure 1 - Coverage across a reference in the autosomal chromosomes for the newly generated fecal samples BF59, BF221, BF270, BF312, and BF315. Plots are generated with qualimap bamqc as described in the Material and Methods section. Each panel refers to one of the five samples as indicated on the top. The vertical lines define the length of the chromosomes. The names of the chromosomes are derived from the NCBI RefSeq nomenclature: NC_044976.1 (chromosome 1), NC_044977.1 (chromosome 2), NC_044978.1 (chromosome 3), NC_044979.1 (chromosome 4), NC_044978.1 (chromosome 5), NC_044981.1 (chromosome 6), NC_044982.1 (chromosome 7), NC_044983.1 (chromosome 8), NC_044984.1 (chromosome 9), NC_044985.1 (chromosome 10), NC_044986.1 (chromosome 11), NC_044987.1 (chromosome 12), NC_044989.1 (chromosome 13), NC_044990.1 (chromosome 14), NC_044990.1 (chromosome 15), NC_044991.1 (chromosome 16), NC_044992.1 (chromosome 17), NC_044993.1 (chromosome 18), NC_044994.1 (chromosome 19), NC_044995.1 (chromosome 20). The figure starts on the previous page.

A) Mt papio phylogeny

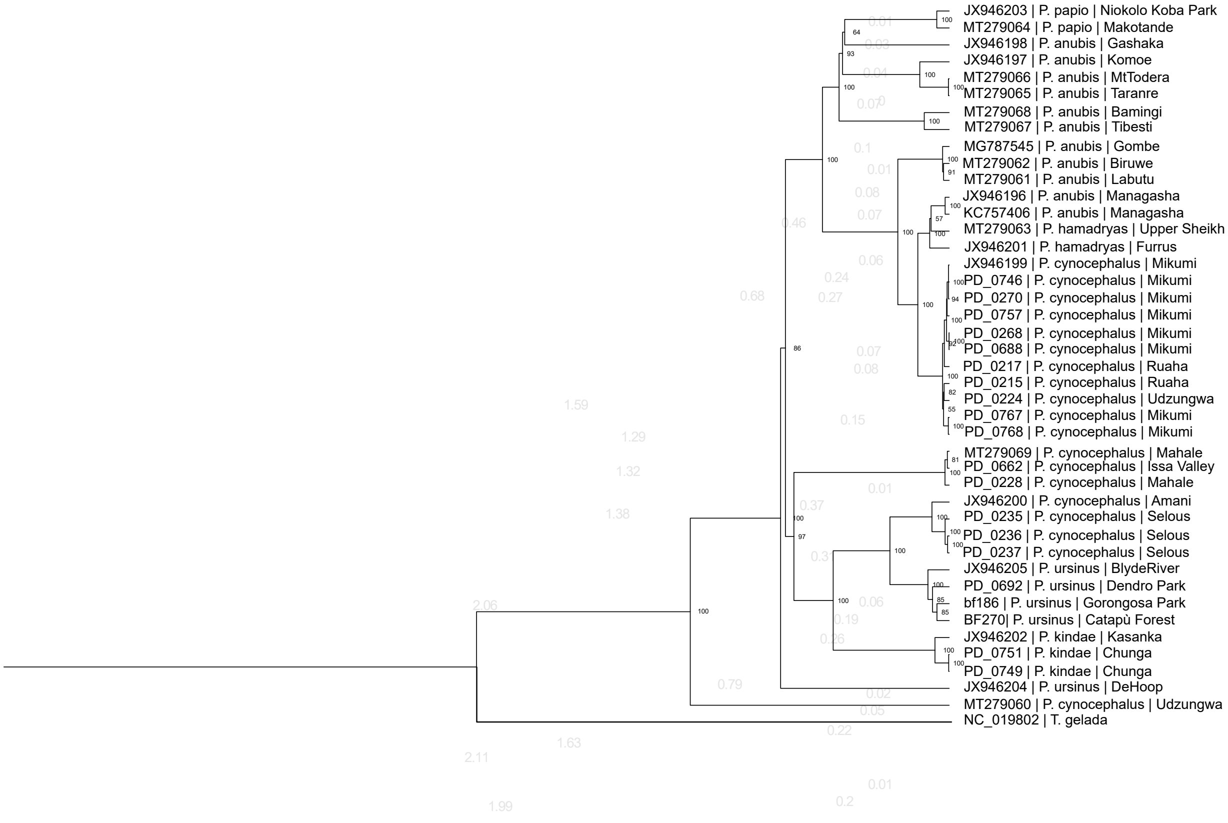

B) Y papio phylogeny

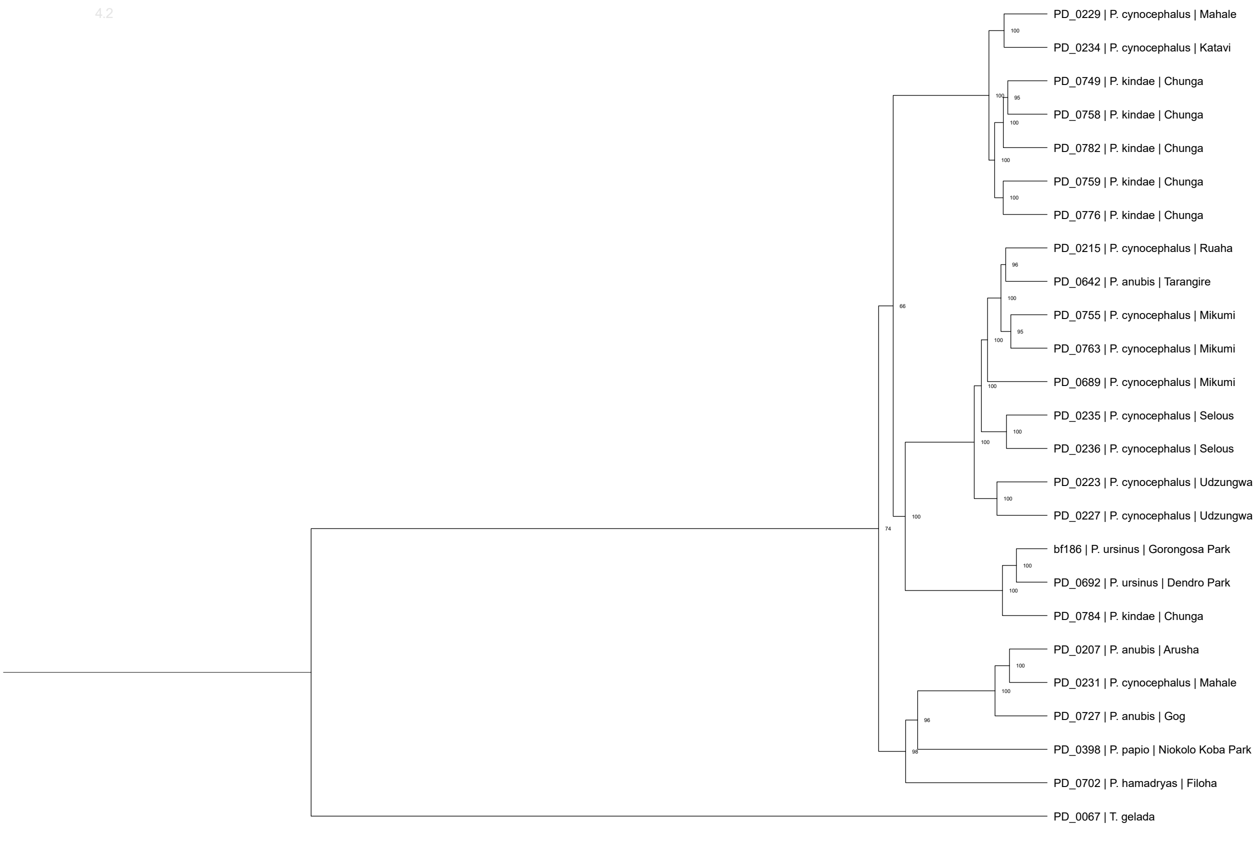

SFigure 2 - Uncollapsed phylogenetic trees using uniparental markers. A) mitochondrial DNA phylogenetic tree based on 43 mitogenomes, on which Figure 2A is based. B) Y chromosome DNA phylogenetic tree based on 25 samples, on which Figure 2B is based. Labels are ordered as follows: sequence/individual ID, species, sampling location (whenever available).

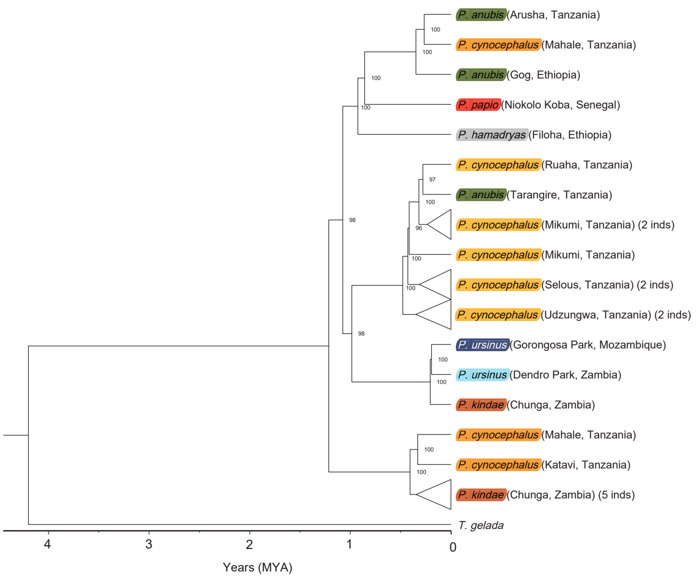

SFigure 3 - Y chromosome tree based on 25 samples mapped on M. mulatta reference genome. For *P. cynocephalus*, eastern and western locations as in Figure 1. Labels are ordered as follows: species (P: *Papio*; T: *Theropithecus*), sampling location (whenever available), and country of origin (CAR: Central African Republic; DRC: Democratic Republic of Congo). For collapsed clades, the number of individuals is also reported. Bootstrap values over 1000 resamplings are reported near nodes.

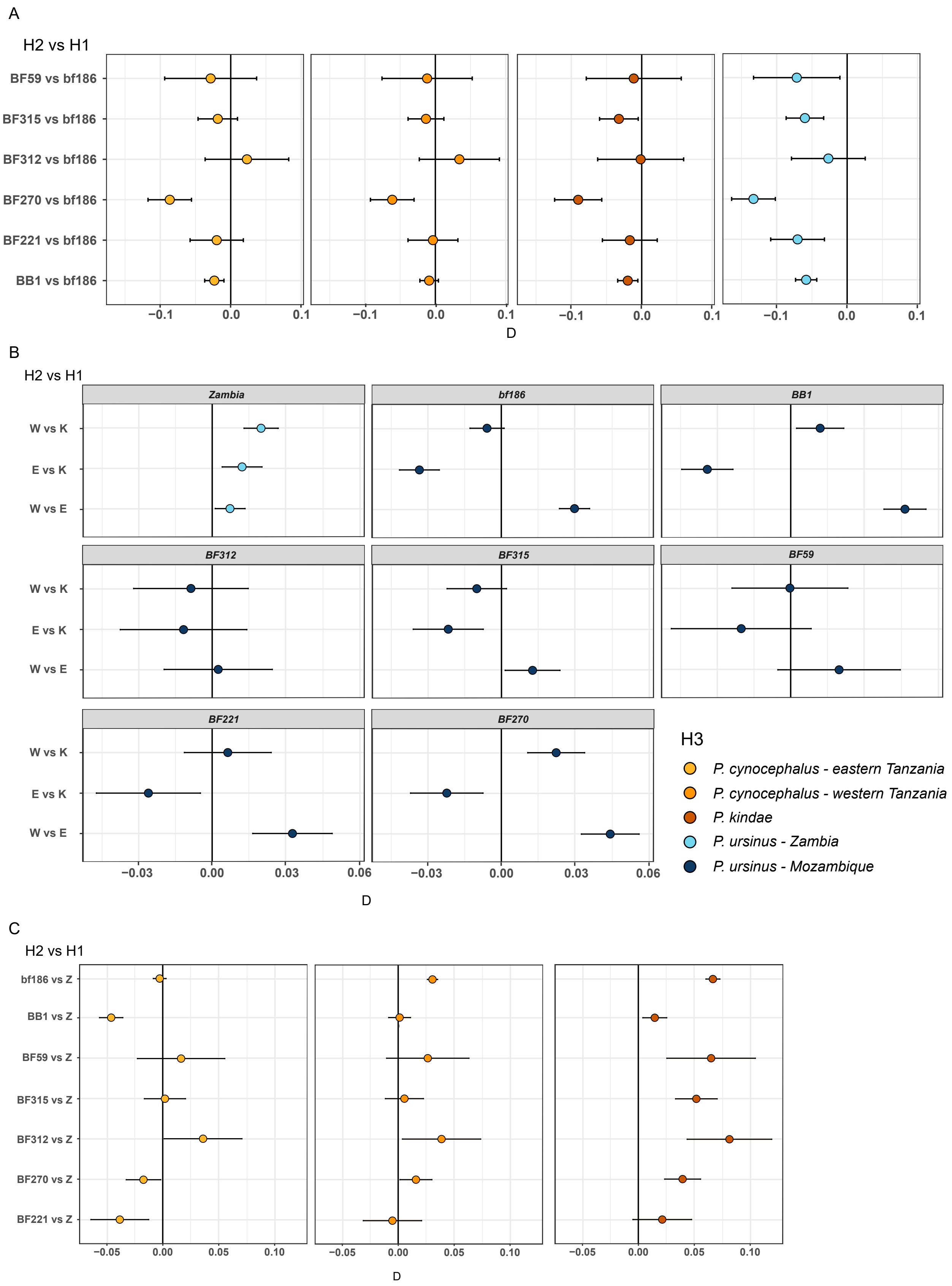

SFigure 4 - A) D-statistics comparing the high coverage (bf186 as H1) and the low coverage (the newly generated BF59, BF221, BF270, BF312, BF315 and the published BB1 as H2) individuals from Gorongosa National Park to neighbouring taxa (*P. cynocephalus* from eastern Tanzania, *P. cynocephalus* from western Tanzania, *P. kindae* and *P. ursinus* from Zambia; H3). D-statistics calculated using only the high-coverage sample (bf186) for the Mozambican population B) comparing neighbouring species (*P. cynocephalus* from eastern Tanzania, *P. cynocephalus* from western Tanzania and *P. kindae* as H2 and H1) to chacma (Mozambique samples and Zambia population as H3); C) comparing chacma populations (same as B; as H2 and H1) to neighbouring species (same as B; as H3). In A, B and C the bars show the extension of three standard deviations and the colours refer to the taxon used as H3 as indicated in the legend; in B and C the H1 and H2 labels refer to *P. cynocephalus* from eastern (E) Tanzania, *P. cynocephalus* from western Tanzania (W), *P. kindae* (K), and *P. ursinus* from Zambia (Z). For positive values, the signal of gene-flow is between H3 and H1, while for negative values the signal is between H3 and H2.

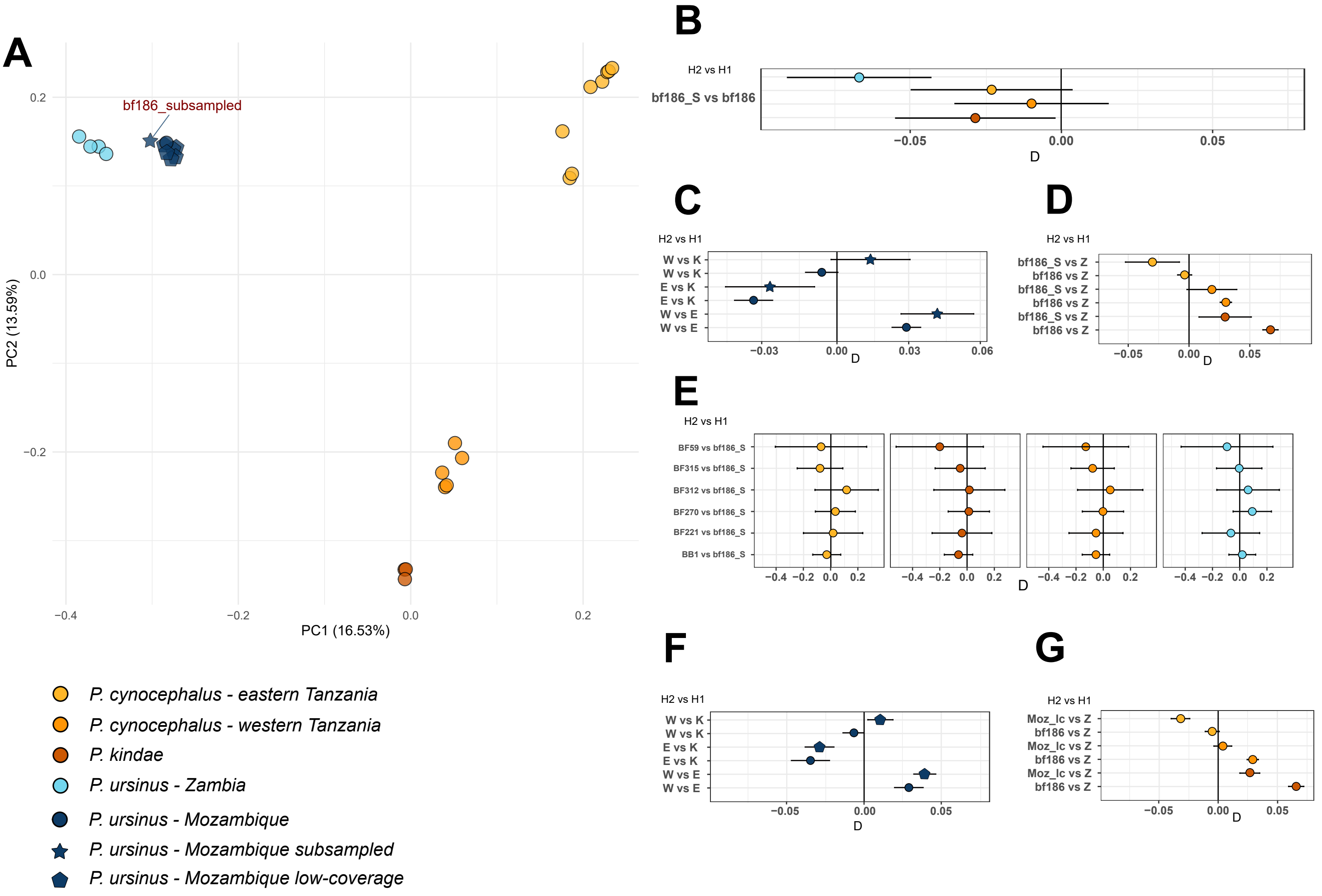

SFigure 5 - bf186 subsampled A) PCA of autosomal genome-wide markers, bf186 subsampled is highlighted; B) D-statistics comparing the subsampled high coverage (bf186_S as H2) and the high coverage sample (bf186 as H1) from Gorongosa National Park to neighbouring species (*P. cynocephalus* from eastern Tanzania, *P. cynocephalus* from western Tanzania, *P. kindae* and *P. ursinus* from Zambia as H3); C) comparing neighbouring species (same as B; as H2 and H1) to chacma bf186 subsampled (and the full-coverage sample) as H3; D) comparing bf186 subsampled and bf186 full-coverage (as H2) and Zambia (as H1) to neighbouring species (same as B; as H3); E) D-statistics comparing the subsampled high coverage (bf186_S as H1) and the low coverage (BF59, BF221, BF270, BF312, BF315, BB1 as H2) individuals from Gorongosa National Park to neighbouring species (P. cynocephalus from eastern Tanzania, P. cynocephalus from western Tanzania, P. kindae and P. ursinus from Zambia as H3); F) comparing neighbouring species (same as B; as H2 and H1) to Mozambique chacma low-coverage as population (and the high-coverage sample) as H3; G) comparing Mozambique low-coverage as population (Moz_lc) and bf186 (as H2) and Zambia (as H1) to neighbouring species (same as B; as H3). In B, C, D, E, F and G the bars show the extension of three standard deviations and the colours refer to the taxon used as H3 as indicated in the legend; in C and D the H1 and H2 labels refer to *P. cynocephalus* from eastern (E) Tanzania, *P. cynocephalus* from western Tanzania (W), *P. kindae* (K), and *P. ursinus* from Zambia (Z). For positive values, the signal of gene-flow is between H3 and H1, while for negative values the signal is between H3 and H2.

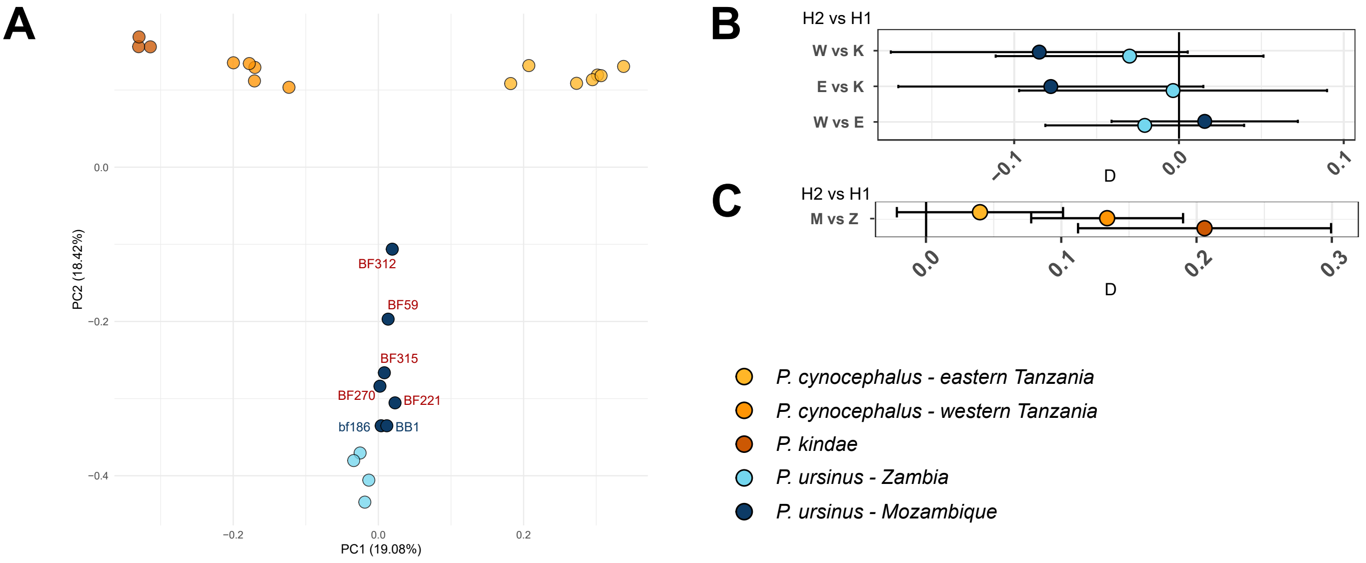

SFigure 6 - Analysis of X-chromosome variants A) PCA of X-chromosome markers; B) D-statistics comparing neighbouring species (*P. cynocephalus* from eastern Tanzania, *P. cynocephalus* from western Tanzania and *P. kindae as H2 and H1) to* chacma pop*ulations (Mozambique and Zambia as H3). C) D-statistics comparing chacma populations (same as B; as H2 and* H1) to neighbouring species (same as B; as H3). In B and C, the bars show the extension of three standard deviations and the colours refer to the taxon used as H3 as indicated in the legend; In B and C, the H1 and H2 labels refer to *P. cynocephalus* from eastern (E) Tanzania, *P. cynocephalus* from western Tanzania (W), *P. kindae* (K), *P. ursinus* from Zambia (Z) and *P. ursinus* from Mozambique (M). For positive values, the signal of gene-flow is between H3 and H1, while for negative values the signal is between H3 and H2.
